# Twisted Fate: A Case Study of Postmenopausal Ovarian Torsion With Gangrene

**DOI:** 10.7759/cureus.68040

**Published:** 2024-08-28

**Authors:** Ishaan M Deshmukh, Prakriti Sharma, Pratyaksh Chhabra, Prerna Sahu, Revat J Meshram

**Affiliations:** 1 Obstetrics and Gynaecology, Jawaharlal Nehru Medical College, Datta Meghe Institute of Higher Education and Research, Wardha, IND; 2 Paediatrics, Jawaharlal Nehru Medical College, Datta Meghe Institute of Higher Education and Research, Wardha, IND

**Keywords:** oophorectomy, laparotomy, transvaginal ultrasound, gangrene, ovarian torsion

## Abstract

An emergency medical evaluation for ovarian torsion should be conducted on any female who complains of stomach pain. More women in the reproductive age range experience ovarian torsion. Ovarian torsion presenting postmenopausal is an uncommon occurrence. Torsion of ovaries can occur alone or with torsion of the fallopian tubes. Pain is the main presenting complaint, which may be accompanied by swelling, nausea, etc. In this instance, we have a 52-year-old woman who complained of lower abdominal pain on the right side. A transvaginal ultrasonography (USG) was done, and torsion of the right ovary was found. On further exploration, it was found to be gangrenous.

## Introduction

The adnexa, or network of structures, encircles the uterus. The ovaries and fallopian tubes are involved in the adnexa. Each side of the uterus has one ovary. The utero ovarian ligament is unique and holds the ovaries in place [1]. A complete or partial rotation of the pedicle causes ischemia in the ovary. While torsions afflict both tissues more frequently, there are fewer solitary torsions involving the fallopian tube or the ovary [2]. When the ovary twists over its supporting ligaments, it is said to have undergone torsion. Adnexal torsion is the term used to describe the twisting of the fallopian tube in tandem with the ovary. Of women who have undergone prior surgical treatment for adnexal masses or cysts, 2-15% develop ovarian torsion [3].

About 3% of gynecologic crises are ovarian torsion instances, and in 80% of these cases, there is also an ovarian mass measuring at least 5 cm at the time of presentation. While it can manifest in any age of the female, postmenopausal-age ladies are less likely to experience it than reproductive-age females [4]. The ovary physically twists at an attachment point, obstructing the lymphatic, venous, and arterial systems. This leads to ovarian thrombosis, edema, necrosis, and infarction in the pathogenesis of torsion. While the clinical image of “acute-onset pelvic pain in a childbearing age female” is commonly painted in traditional textbook presentations of ovarian torsion, real-world patients frequently present with considerably more subtle and complex symptomatology, which can provide considerable diagnosis difficulty [5]. To demonstrate that adnexal torsion can occur at any age and that having an ovarian tumor or cyst predisposes one to adnexal torsion, this report describes a rare case of adnexal torsion in a postmenopausal woman [6].

## Case presentation

A 52-year-old, para 2 live 2, postmenopausal woman married for 28 years, presented to the emergency department with a complaint of pain in the right flank for the last 15 days. The pain was dull and aching, followed by swelling. She had nausea and vomiting for four days. She also experienced abdominal fullness, which caused loss of appetite and sometimes difficulty in sleeping due to discomfort. She had no urinary symptoms or no fever. She had a history of similar episodes two months earlier when she visited a local hospital, and the doctor prescribed her analgesic medication for seven days. After taking the medication, her pain was relieved for some days until she presented with current complaints.

On examination, her build was average, and she weighed 60 kg. The patient was afebrile to touch and had a blood pressure of 130/90 mmHg. The cardiovascular system and respiratory systems were normal. On abdominal examination, she had a palpable abdominal mass and tenderness on the right side. The pain did not radiate to any other part of the body. She was advised to have a transvaginal ultrasonography (USG). The scan showed a normal-sized left ovary, measuring 3.4 cm × 2.5 cm × 1.5 cm. On the right side, a torsion in the ovary was seen, and the size of the ovary measured 5 cm × 3.5 cm × 2.9 cm. Doppler was also done, and absent flow was observed. The patient was then told about the reports and was guided on further management. Video 1 shows the transvaginal USG, showing a twisted pedicle that is suggestive of torsion of the ovary.

**Video 1:** Twisted pedicle suggesting torsion in the ovary

Management

**Figure 1:**
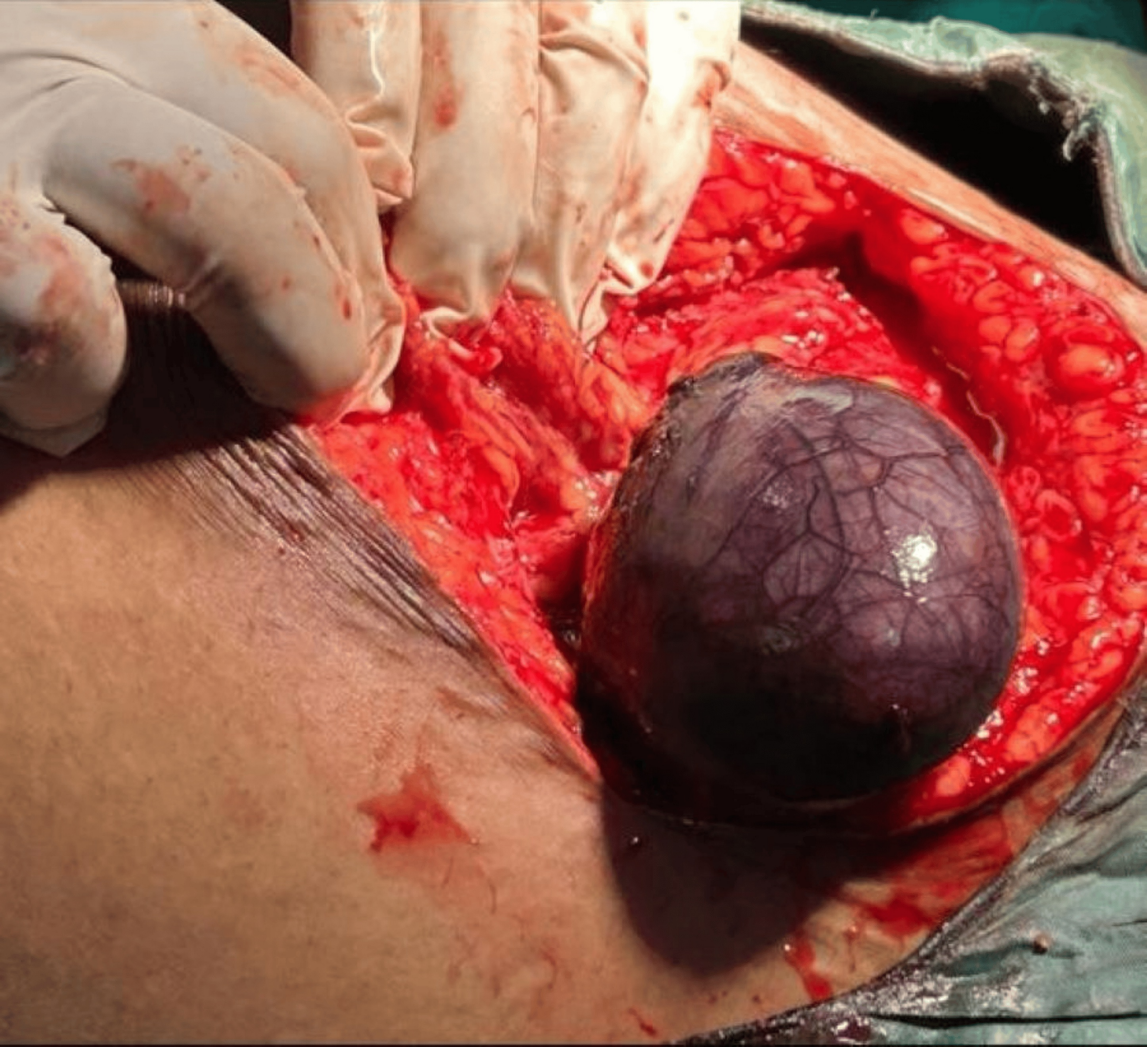
Gangrenous ovary visualized on laparotomy

After informing the patient about the report, she was told about the further management that would be done. She was informed that an exploratory laparotomy would be done and possible defects would be looked out. Mostly in cases of ovarian torsion, detorsion of ovaries is performed so as to preserve the function and the organ itself, and she was informed of this. She was also informed that during the procedure, if we find any gangrenous presentation in the ovary, then an excision of the ovary would be necessary. An intravenous line was started, and she was put under general anesthesia. A vertical abdominal incision was made down the middle of the abdomen along the linea alba, and the ovary was observed. On looking at the ovary, it was found to be blackish purple in color, which indicated gangrenous pathology (Figure 1). It was decided to perform a unilateral oophorectomy (surgical removal of the ovary). The incision was closed, and she was advised on further care. 

## Discussion

The instances that are being given highlight how all female patients experiencing stomach pain should have ovarian torsion considered as a possible diagnosis, especially by emergency medicine and other healthcare professionals. They also demonstrate how even patients with unusual presentations need workups to make this diagnosis, underscoring the care guidelines for ovarian torsions. It is crucial to keep in mind that any female patient with ovaries may exhibit symptoms of ovarian torsion. It is still a reasonable differential diagnosis for any girl experiencing stomach discomfort. Other risk factors for ovarian cyst torsion include pregnancy, ovarian stimulation, a history of abdominal surgery, and tubal ligation [7].

The patient in our case was a 52-year-old postmenopausal para 2 live 2 woman who had abdominal pain for 15 days along with indications of fullness and decreased appetite. A twisted right ovary was successfully removed during an abdominal hysterectomy and unilateral oophorectomy. Although it can be difficult to diagnose ovarian torsion, it is important to carefully examine the symptoms, particularly if the lower abdomen pain appears suddenly. Pelvic USG is useful in identifying ovarian cysts. The main method for identifying and treating ovarian torsion is surgery [8]. Oophorectomy, ovarian cystectomy, and detorsion are examples of preferred treatment choices. With an incidence of 2.7%, adnexal torsion is most common in the reproductive age group but less common in postmenopausal women. Known as an ovarian cyst torsion, the ovarian vascular pedicle can rotate fully or partially, preventing blood flow. Ovarian cyst torsion can affect anyone at any age, but it is more common in women in their 20s and 30s. Treatment options for premenarchal girls with adnexal torsion include detorsion and oophoropexy, which attempt to protect the ovaries [9].

While laparoscopic surgery is more commonly performed on women of reproductive age, laparotomy is more commonly performed on postmenopausal women. Since postmenopausal women rarely experience adnexal torsion, the diagnosis is frequently overlooked [10]. When the diagnosis is delayed, it can lead to worse outcomes since the patient’s history and physical examination are less trustworthy. Bimanual examinations, blood tests, and imaging studies, such as ultrasound, computed tomography, or magnetic resonance imaging of the pelvic organs, can be used by doctors to identify the illness. The size of the cyst, the tools at hand, and the experience level of the physician determine the course of treatment. Using a laparoscopic technique, a simple detorsion was initially decided upon for this patient [11].

## Conclusions

Adnexal/ovarian torsion is difficult to diagnose. The initial diagnostic method for identifying an adnexal mass or cyst, which increases the risk of adnexal torsion, is USG. Surgery is the primary treatment option for adnexal/ovarian torsion, and depending on the patient’s age and potential for fertility, this may involve either radical (oophorectomy or adnexectomy) or conservative (ovarian cystectomy and/or detorsion). When there is a gangrenous appearance, the ovary must be surgically removed, but in most non-gangrenous situations, detorsion is considered to retain the structure and function of the ovary.
